# Osteoblastic Differentiation on Graphene Oxide-Functionalized Titanium Surfaces: An In Vitro Study

**DOI:** 10.3390/nano10040654

**Published:** 2020-04-01

**Authors:** Roberta Di Carlo, Antonello Di Crescenzo, Serena Pilato, Alessia Ventrella, Adriano Piattelli, Lucia Recinella, Annalisa Chiavaroli, Silvia Giordani, Michele Baldrighi, Adalberto Camisasca, Barbara Zavan, Mirella Falconi, Amelia Cataldi, Antonella Fontana, Susi Zara

**Affiliations:** 1 Department of Medical, Oral and Biotechnological Sciences, University “G. d’Annunzio” of Chieti-Pescara, via dei Vestini 31, 66100 Chieti, Italy; 2 Department of Pharmacy, University “G. d’Annunzio” of Chieti-Pescara, via dei Vestini 31, 66100 Chieti, Italy; 3 School of Chemical Sciences, Dublin City University, Glasnevin, D09 E432 Dublin 9, Ireland; 4 Nano Carbon Materials, Italian Institute of Technology, via Morego 30, 16163 Genova, Italy; 5 Medical Science Department, University of Ferrara, via Aldo Moro 8, 44121 Ferrara, Italy; 6 Department of Biomedical and Neuromotor Sciences, University of Bologna, via Irnerio 48, 40126 Bologna, Italy

**Keywords:** titanium disc, surface functionalization, graphene oxide, dental pulp stem cells, osteoblastic differentiation

## Abstract

Background: Titanium implant surfaces are continuously modified to improve biocompatibility and to promote osteointegration. Graphene oxide (GO) has been successfully used to ameliorate biomaterial performances, in terms of implant integration with host tissue. The aim of this study is to evaluate the Dental Pulp Stem Cells (DPSCs) viability, cytotoxic response, and osteogenic differentiation capability in the presence of GO-coated titanium surfaces. Methods: Two titanium discs types, machined (control, Crtl) and sandblasted and acid-etched (test, Test) discs, were covalently functionalized with GO. The ability of the GO-functionalized substrates to allow the proliferation and differentiation of DPSCs, as well as their cytotoxic potential, were assessed. Results: The functionalization procedures provide a homogeneous coating with GO of the titanium surface in both control and test substrates, with unchanged surface roughness with respect to the untreated surfaces. All samples show the deposition of extracellular matrix, more pronounced in the test and GO-functionalized test discs. GO-functionalized test samples evidenced a significant viability, with no cytotoxic response and a remarkable early stage proliferation of DPSCs cells, followed by their successful differentiation into osteoblasts. Conclusions: The described protocol of GO-functionalization provides a novel not cytotoxic biomaterial that is able to stimulate cell viability and that better and more quickly induces osteogenic differentiation with respect to simple titanium discs. Our findings pave the way to exploit this GO-functionalization protocol for the production of novel dental implant materials that display improved integration with the host tissue.

## 1. Introduction

Commercially pure titanium (Ti) is a substrate with excellent physico-chemical and mechanical properties, such as high biocompatibility, low density, resistance to corrosion, and durability [1]. For all these reasons, Ti and its alloys are generally considered the most useful materials for the development of prosthetic implants [2]. The close contact between bone tissue and dental implants is a fundamental prerequisite of osseointegration, and the bone cells adhesion on Ti implants strictly depends on implant surface properties, such as roughness, topography, chemistry, charge, and wettability [3]. As the surface topography exerts a significant impact on cell–implant interactions, chemical and physical strategies are frequently employed to improve its properties. This processing positively influences the implant integration with the host tissue, and consequently the clinical outcome. Rougher implant surfaces provide better biomechanical stability at the bone/implant area than smooth machined surfaces, by establishing a greater contact with the host tissue [4,5]. Several different surface modifications were found to induce a significant roughness increase of the titanium surfaces [6], such as treatment with inorganic ions such as magnesium and calcium [7,8,9]. 

Besides topography, surface coating of dental biomaterials with bioactive molecules was found to be responsible for both increased osteoblasts proliferation and differentiation rate [10].

Graphene and its derivatives are a class of compounds that are attracting a lot of attention for their good biocompatibility [11,12]. Graphene is a flat monolayer of sp^2^-hybridized carbon atoms, arranged in a two-dimensional honeycomb lattice, with extraordinary electrical, thermal, physical, and mechanical properties. In addition, it is optically transparent. It behaves as an impermeable membrane and is chemically stable [2]. For these features, it is a good candidate for various applications in different fields [13,14,15,16]. In particular, the world of dentistry is approaching graphene-based nanomaterials as scaffolds for tissue engineering, due to the possibility of graphene and its derivatives to be incorporated into different biomaterials, despite that their use in regenerative medicine is still at an early stage [17]. Graphene can be chemically modified to obtain its oxidized derivative, namely graphene oxide (GO), which has been actively investigated for applications in the biomedical field due to its solubility in water, solution spreadability over surfaces, and its reactive oxygen functional groups that allow easy further functionalization or grafting. Studies have proven that graphene and GO are highly biocompatible and characterized by low toxicity levels, thus, allowing their use as support for tissue regeneration, cell growth, and cell differentiation [5,18,19,20]. Since a few studies have highlighted that graphene and GO sheets may provide biocompatibility issues depending on the degree of exfoliation and dimensions of sheets [21,22], in the present study we have used a commercial GO aqueous dispersion that ensures a reproducibility of GO features, and that have evidenced no toxicity on dental pulp stem cells [10]. To date, only very few studies have highlighted the improvement of biocompatibility and osteogenic differentiation of GO-deposited titanium surfaces with respect to the pure titanium samples, and the majority of them investigated electrophoretic deposited [23] or physically deposited GO [24,25]. In the present study, we investigated covalently GO-functionalized titanium since, to the best of our knowledge, studies involving materials obtained through chemical functionalization of GO on titanium surfaces are really limited [26,27] and devoted to other aspects and applications [27,28]. 

Stem cells are a promising tool for tissue regeneration, thanks to their characteristics of proliferation, differentiation, and plasticity. Undifferentiated and highly clonogenic cells may be isolated from the dental pulp, periodontal ligament, and dental papilla [29]. In particular, dental pulp is an appreciable source of dental pulp mesenchymal stem cells (DPSCs) because of the simple surgical sample’s withdrawal, extremely low morbidity of the anatomical site after pulp harvesting, and the easy extraction of stem cells from harvested samples. DPSCs, whose embryological derivation is from the neural crest [30], are also well known for their extensive differentiation ability, good interaction with biomaterials [31], and ability to differentiate into mature odontoblasts/osteoblasts producing mineralized matrix [32].

The aim of this study was therefore to functionalize machined titanium discs (control group, Crtl) and sandblasted and acid-etched (double attack) titanium discs (test group, Test) with GO, to investigate the GO capability to promote DPSCs proliferation and differentiation into osteogenic cells lineage. Hence, the first step in this work was to optimize a protocol of covalent functionalization of titanium discs with GO, in order to create a well bound coating of GO and reduce GO leaking in the bulk solution. Secondly, we investigated the biocompatibility of the GO-enriched titanium discs with DPSCs, and eventually their capacity to promote osteogenic differentiation.

## 2. Materials and Methods

### 2.1. Materials 

Experimental pure (grade IV) titanium discs (cut at the elected dimensions of 5 mm diameter and 2 mm thickness, 177 ± 20  mg weight) (Implacil De Bortoli-Dental Product, São Paulo, Brazil) were used. Two different Ti discs were investigated: control Ti discs (Ctrl) were discs with a machined surface; test discs (Test) were sandblasted with titanium powder, then cleaned with purified water, enzymatic detergent, acetone, citric acid (double acid attack), and alcohol. A hundred and twenty titanium discs for each type were used. Graphene oxide (GO) aqueous solution was obtained as a commercial sample from Graphenea, Donostia-San Sebastian, Spain and already characterized by the manufacturing company in terms of exfoliation, size, and oxidation degree. Unless otherwise specified, reagents were used as received and were purchased from Merck, Darmstadt, Germany.

### 2.2. Functionalization of Titanium Discs 

Both the two types of Ti implants were surface modified with GO to yield Ctrl + GO and Test + GO samples. Each type of GO-functionalized Ti implant was compared with the corresponding non functionalized sample. In order to obtain GO-coated titanium discs, a protocol of covalent functionalization was optimized. The first step was the activation of the Ti discs. Control samples were activated by immersing them in a piranha solution (3:1 vol/vol H_2_SO_4_/H_2_O_2_) for 1 h, while test samples implants were activated using an UV/ozone lamp (PSD-UV4 Novascan UV Ozone System Base Model, Novascan Technologies, Boone, IA, US) for 30 min on each side. The different protocol of activation was due to the fact that doubly etched Titanium discs (Test) did not stand piranha solution treatment, probably because of residual citric acid decomposition. After activation, the samples were rinsed with deionized water and soaked in ethanol for five min. The second step comprised the immersion of 10 discs (ca. 177 ± 20 mg each) in 2% (3-amminopropyl) triethoxysilane (APTES) ethanol solution (5 mL) for 40 min. After washing the discs with pure ethanol to remove unabsorbed APTES, the so-obtained aminosilane-functionalized discs were left to dry again at room temperature. The last step consisted of pouring an aqueous solution (50 µL) of 2 mg mL^−1^ GO on the APTES-functionalized discs for 5 min, and spin coating each disc at 100 rpm for 2 s, and at 2000 rpm for 31 s, in order to obtain a thin layer of GO on the disc surface. The GO-coated implants were left to dry for 1 h, before washing with pure ethanol to remove the unbound material. Samples were exposed to UV light for 1 h, and then transferred in 48-well trays for the in vitro experiments.

### 2.3. Raman Analyses 

Raman spectra were acquired by means of a Horiba Jobin Yvon LabRam HR 800 Raman microscope (Horiba Ltd, Kyoto, Japan), using the built-in 632 nm laser as light source. All the spectra were normalized, with respect to the G band, for comparison of the Raman intensities.

### 2.4. Atomic Force Microscopy (AFM) and Scanning Electron Microscopy (SEM) Analyses

GO-coated discs were characterized with an atomic force microscope (AFM) by using the Multimode 8 AFM (Bruker, Billerica, MA, US) with Nanoscope V controller, imaging mode Scan Asyst^TM^ in air using the silicon tip RTESP-300 (cantilever resonance frequency 300 kHz and nominal elastic constant 40 N/m) with a tip radius of 8 nm, in order to analyze properties such as topography and peak force error across a scan size area of 10 μm × 10 μm.

For SEM measurements, samples were fixed with 1.25% glutaraldheyde in 0.1 M cacodylate buffer for 30 min, before dehydrating them through alcohol series. Then, samples were dried with hexamethyldisilazane, followed by gold-coating. Micrographs were obtained at 15 kV on a compact desktop Phenom XL SEM microscope (Thermo Fisher Scientific, Eindhoven, The Netherlands).

### 2.5. Isolation and Cultivation of DPSCs 

The project obtained the approval of the Local Ethical Committee of the University of Chieti (approval number 1173, date of approval 03/31/2016), in accordance with the Declaration of Helsinki. Dental pulp samples were obtained from third molars extracted from young adults (the age range was 18–28 years) who underwent orthodontic treatments. Informed consent was obtained from all the participants. Only impacted teeth without dental pathologies were included in the study. After the extraction, all the remaining soft tissues were mechanically removed and processed, as reported in our previous work [7]. 

The dental pulp was washed with phosphate-buffered saline (PBS), kept in Minimum Essential Medium Eagle, alpha Modification (α-MEM, Sigma-Aldrich, St. Louis, MO, US) with the addition of 10% of Foetal Bovine Serum (FBS) and 1% of penicillin/streptavidin (EuroClone S.p.A, Milan, Italy), and delivered to the laboratory for stem cells isolation [7]. When cell culture reached sub-confluence (80–90% of surface area), they were subcultured. Antigen expression of CD29, CD45, CD105, CD73 CD90, and SSEA-4 was checked by flow cytometry [7].

### 2.6. DPSCs Cultivation on Titanium Discs 

DPSCs from the fourth or fifth passage were seeded on Ti discs at a density of 10,000 cells/cm^2^ and cultivated for 24 h in α-MEM, supplemented with 10% FBS and 1% penicillin/streptavidin. After 24 h, the standard medium was removed and replaced by a differentiating medium containing the differentiating factors (10 nM dexamethasone, 0.2 mM ascorbic acid, 10 mM β-glycerophosphate). Cells were cultured in differentiating medium for up to 28 days by refreshing it two times per week. At the established times, cells were harvested and processed for the required analyses. 

### 2.7. Scanning Electron Microscopy (SEM) Analysis of Cultured Cells 

Samples were fixed with 2.5% glutaraldehyde in 0.1 M cacodylate buffer for 1 h before processing with either hexamethyldisilazane or critical-point drying, followed by gold-palladium coating. All micrographs were obtained at 20 kV on a JEOL 6360LV SEM microscope (JEOL, Tokyo, Japan).

### 2.8. MTT Assay 

3-[4,5-dimethyl-thiazol-2-yl-]-2,5-diphenyl tetrazolium bromide (MTT) (Sigma Aldrich, Saint Louis, MO, US) analysis was performed after 3, 7, 14, 21, and 28 days of DPSCs culture on Ti discs. At the established times, the medium was replaced by medium containing 0.5 mg/mL MTT and probed with cells for 5 h at 37 °C. In order to solubilize salts, the plate was incubated in DMSO (dimethylsulfoxide) for 30 min at 37 °C. Spectrophotometric reading of the optical density was performed at 570 nm by means of a microplate reader (Multiskan GO, Thermo Scientific, Vantaa, Finland). Values obtained in the absence of cells were considered as background.

### 2.9. Lactate Dehydrogenase (LDH) Cytotoxicity Assay 

To evaluate membrane integrity of DPSCs, LDH leakage into the medium was quantified by using a CytoTox 96 non-radioactive cytotoxicity assay (Promega, Madison, WI, US), as recommended by the manufacturer, after 3, 7, 14, 21, and 28 days of culture on titanium discs. In each well, the measured LDH leakage in the supernatant was normalized to the lysis value, obtained by adding a lysis solution to another well of the same experimental condition.

### 2.10. RNA Extraction 

Total RNA was extracted using TRI Reagent (Sigma-Aldrich, St. Louis, MO, US). Briefly, cells attached to membranes in each plate well were suspended in 500 µL of TRI Reagent. This suspension was centrifuged at 10,000 rpm for 10 min at 4 °C to remove the insoluble material. The supernatant was added to 100 µL of chloroform, then shaken vigorously, incubated on ice for 15 min, and centrifuged at 13,200 rpm for 20 min at 4 °C. RNA in aqueous phase was precipitated with 250 µL of isopropanol, stored for 30 min at −20 °C, and pelleted by centrifugation at 13,200 rpm for 20 min at 4 °C. The RNA pellet was washed with 500 µL of 75% ethanol, air dried, and resuspended in RNase-free water. 

Contaminating DNA was removed using DNA-free kit (Life Technologies, Carlsbad, CA, US) according to the manufacturer’s instructions. RNA concentration was determined by spectrophotometer readings (BioPhotometer, Eppendorf, Hamburg, Germany) at 260 nm, and its purity was assessed by the ratio at 260 and 280 nm readings. To evaluate the quality of extracted RNA, the samples were tested by electrophoresis through agarose gels and visualized by staining with ethidium bromide, under UV light.

### 2.11. Reverse Transcription (RT) and Real-Time RT-Polymerase Chain Reaction (Real-Time RT-PCR) 

A high capacity cDNA Reverse Transcription kit (Life Technologies Italia, Monza, Italy) was used to reverse transcribe 1 µg of RNA in a reaction volume of 20 µL. Reactions were incubated in a 2720 Thermal Cycler (Life Technologies Italia, Monza Italy), initially at 25 °C for 10 min, then at 37 °C for 2 h, and finally at 85 °C for 5 min.

Gene expression was determined by quantitative PCR, using TaqMan probe-based chemistry. Reactions were performed in 96-well plates on an ABI PRISM 7900 HT Fast Real-Time PCR System (Life Technologies Italia, Monza, Italy). TaqMan probes and PCR primers were obtained from Life Technologies (TaqMan Gene Expression Assays (20X) and are all listed in Table 1. Glyceraldehyde-3-phosphate dehydrogenase (GAPDH) (Life Technologies Italia, Monza, Italy, Part No. 4333764 F) was used as the housekeeping gene. Each amplification reaction was performed with 10 µL of TaqMan Fast Universal PCR Master Mix (2X), No AmpErase UNG (Life Technologies Italia, Monza, Italy), 1 µL of primer-probe mixture, 1 µL of cDNA, and 8 µL of nuclease-free water. No-template control was used to check for contamination. A reverse transcriptase, minus control, was included for SP7 gene Assay.

Thermal cycling conditions were: 95 °C for 20 s, followed by 40 cycles of amplification at 95 °C for 1 s and 60 °C for 20 s. Real-time PCR analyses were performed in three independent experiments. In each experiment, we included one cDNA sample for each experimental condition. Amplification was carried out in triplicate for each cDNA sample in relation to each of the investigated genes. Sequence Detection System software, ver. 2.3 (Life Technologies Italia, Monza, Italy) elaborated gene expression data. The comparative 2-ΔΔCt method was used to quantify the relative abundance of mRNA (relative quantification).

### 2.12. ELISA Test of PGE2 Secretion 

PGE2 secretion in the culture medium was detected following the instructions provided by the manufacturer protocol. EIA kit (Enzo Life Sciences, Farmingdale, NY, USA) was used to measure PGE2 concentrations. The absorption values were obtained by spectrophotometric reading of plates at 450 nm and 405 nm, by means of a microplate reader (Multiskan GO, Thermo Scientific, MA, USA). PGE2 secretion levels were measured in different wells and normalized for optical density (pg/mL/OD) as previously determined by MTT assay.

### 2.13. Statistical Analysis

Statistical analysis was performed using GraphPad Prism version 5.01 for Windows (GraphPad Software, San Diego, CA, US). Data were collected from each sample used in the experimental procedure and means ± SD were determined for each experimental group. Gene expression values were analyzed by one-way analysis of variance (ANOVA). A calibrator sample was obviously considered the theoretical mean for the comparison. In vitro data were evaluated with ANOVA test followed by Newman–Keuls post-hoc test. The level of statistical significance was set as *p* values < 0.05.

## 3. Results

The occurrence of GO functionalization in titanium discs was evaluated by Raman spectroscopy. 

**Figure 1:**
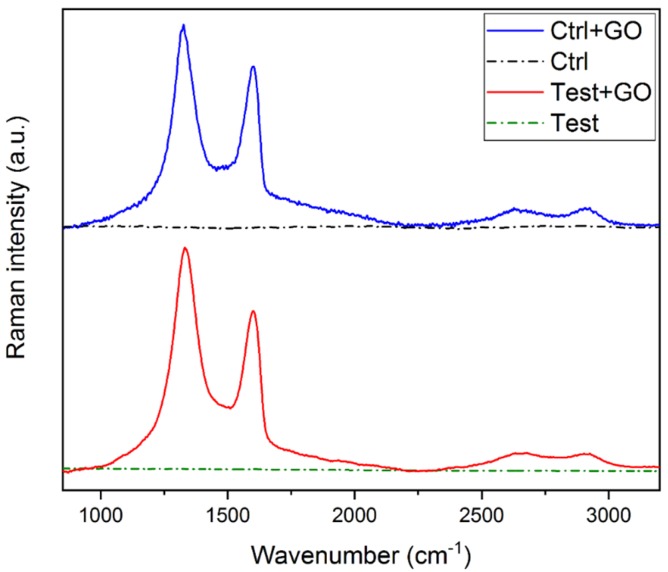
Raman spectra of control + GO (blue line), control (black dash dotted line), test + GO (red line), and test (green dash dotted line). Control + GO and test + GO spectra are normalized with respect to the G band.

As depicted in Figure 1, all GO-coated samples displayed the characteristic D and G Raman bands of GO [33], confirming the presence of a GO layer on the Ti discs, in contrast with the unfunctionalized discs (Ctrl and Test) showing no Raman peaks in the 800–3200 cm^−1^ range.

**Figure 2:**
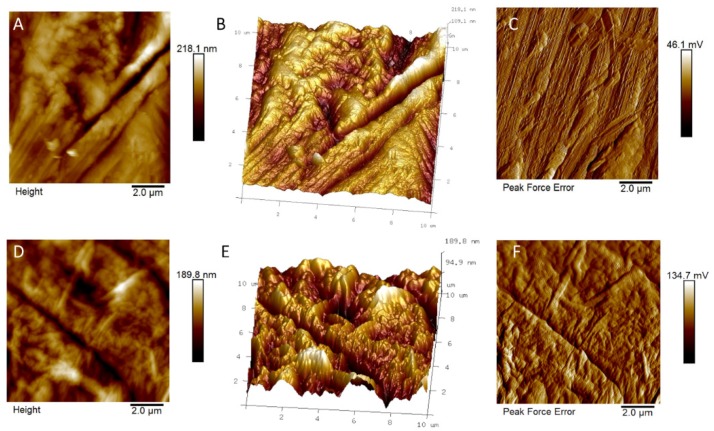
Atomic force microscope (AFM) images of (**A**,**D**) bidimensional and (**B**,**E**) tridimensional topography, as well as (**C**,**F**) peak force error of control (upper line) and control + graphene oxide (GO) discs (lower line).

**Figure 3:**
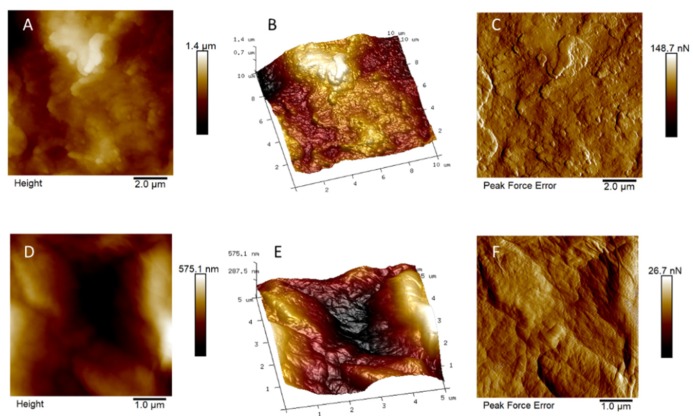
AFM images of (**A**,**D**) bidimensional and (**B**,**E**) tridimensional topography, as well as (**C**,**F**) peak force error of test (upper line) and test + GO discs (lower line).

AFM analyses were performed in order to evaluate the surface roughness of functionalized and not functionalized Ti discs (see Figure 2 and Figure 3). No relevant differences on R_a_ (i.e. the mean of the absolute values of the surface high deviations), R_q_ (i.e. the roughness least square value), R_max_ (i.e. the maximum perpendicular distance between the highest and lowest points in the image following the plane fit), S_dq_ (i.e. the root-mean square of the surface slope), and S_dr_ (i.e. the percentage of additional surface area contributed by the new texture as compared to an ideal plane of the same region) were observed on GO-functionalized samples, with respect to the relevant non-functionalized discs. The average values of five different measurements (see Supplementary material, Table S1) are reported in Table 2.

As shown in Table 2, the test discs surface, either in the absence or in the presence of GO, was rougher than the control discs surface. This was ascribed to the physical/chemical treatments the test discs were subjected to before the functionalization with GO. Indeed, the functionalization with GO did not affect the surface roughness of the Ti discs significantly, in both the control and test samples.

**Figure 4:**
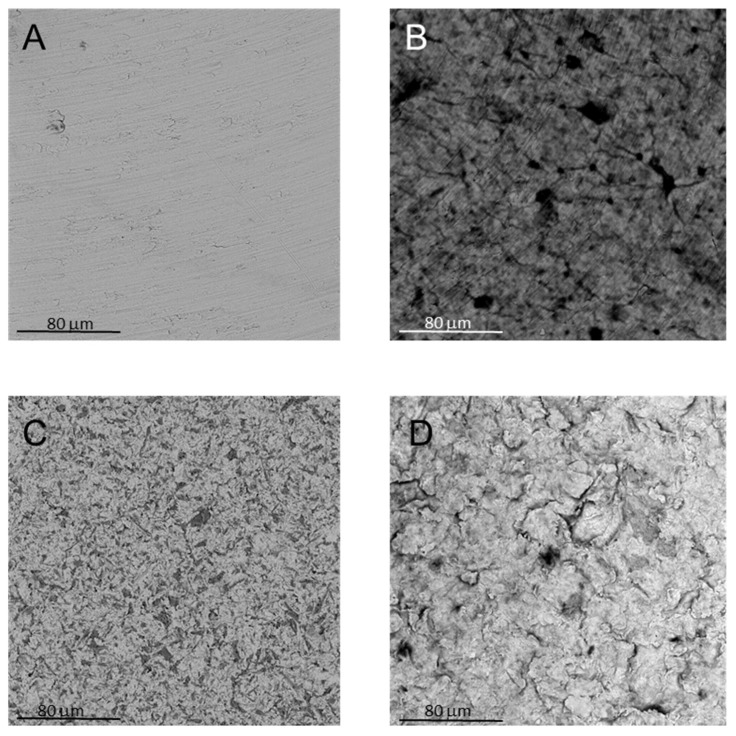
SEM images of (**A**) control; (**B**) control + GO; (**C**) test; and (**D**) test + GO discs.

In agreement with the AFM results, SEM micrographs reported in Figure 4 showed a remarkable difference in the surface topography between the control and test discs, due to the physical/chemical treatments originally performed on Ti discs. Nevertheless, it is possible to notice that GO did alter the disc surface, as flakes of GO could be detected on both types of GO-functionalized discs.

DPSCs were cultured up to 28 days using a differentiating medium on both the control and test discs (raw and GO-functionalized). 

**Figure 5:**
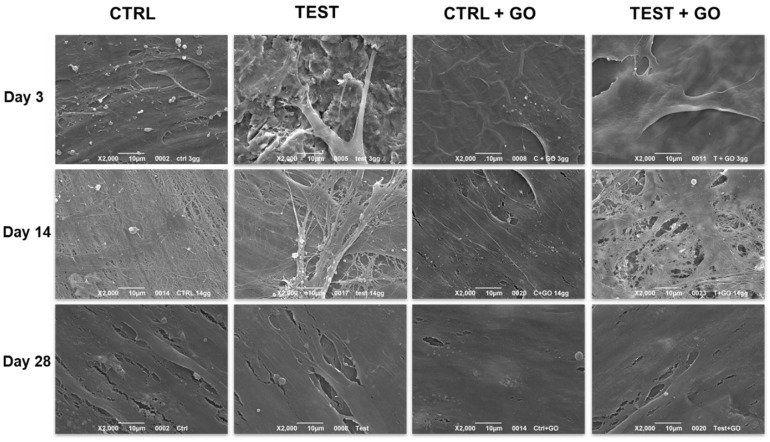
SEM images of DPSCs cultured on control, test, control + GO, and test + GO discs for 3, 14, and 28 days. Magnification 2000×.

The cell-substrate interaction was evaluated by SEM (Figure 5). It could be observed, after three days of culture, that cells were attached on all substrates either in the presence or in the absence of GO. After 14 days of culture, the surface was almost covered by cells, with an appreciable amount of collagen fibers and matrix deposition at all the experimental points. No significant differences in the matrix deposition was found. After 28 days of culture, a complete cell coverage of the surface was evidenced at all the experimental points, with the cells in communication with each other. SEM micrographs showed a high number of specific features (flat and extended bodies, extracellular calcium deposits) that are typical of osteoblasts (Figure 5).

**Figure 6:**
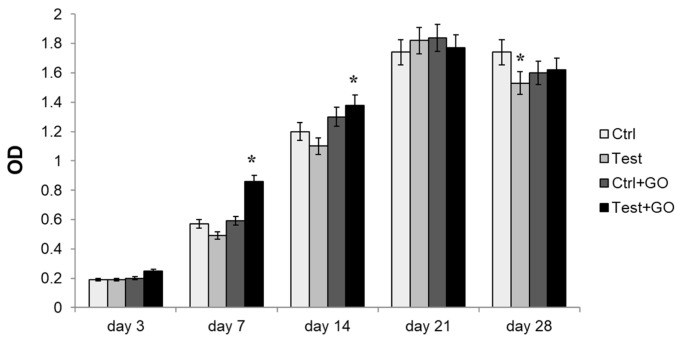
3-[4,5-dimethyl-thiazol-2-yl-]-2,5-diphenyl tetrazolium bromide (MTT ) assay in DPSCs cultured on Ti discs for 3, 7, 14, 21, and 28 days. The histogram represents optical density (OD) values in control, test, control + GO, and test + GO discs. Data shown are the mean (± SD) of three separate experiments. * Day 7: test + GO vs. control, test, control + GO *p* < 0.01. * Day 14: test + GO vs. control, test *p* < 0.05. * Day 28: test vs. control *p* < 0.05.

Metabolic activity was measured by means of MTT test after 3, 7, 14, 21, and 28 days of culture. After seven days of culture, a statistically significant increase in cell metabolic activity was recorded in the test + GO sample, in comparison to all other experimental points. After 14 days of culture, a significant increase in metabolic activity was found in the test + GO sample with respect to both control and test samples, while the increase observed for control + GO with respect to both control and test samples was not statistically significant. After 28 days of culture, a statistically significant decrease in cell metabolic activity could be identified in the test sample with respect to the control (Figure 6).

**Figure 7:**
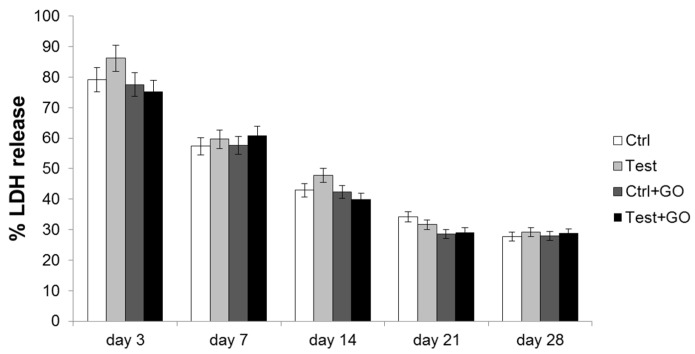
LDH assay of DPSCs cultured on control, test, control + GO, and test + GO discs for 3, 7, 14, 21, and 28 days. LDH released is reported as percentage. Data shown are the mean (± SD) of three separate experiments.

Biomaterial cytotoxicity was measured through lactate dehydrogenase (LDH) assay. After three days of culture, the released LDH% was generally high in all tested experimental samples. Starting from the seventh day of culture, the cytotoxicity level began to decrease from that measured after the third day of culture in all tested experimental points. This trend was maintained over time culture. Moreover, after 3, 14, and 21 days of culture, a decrease of LDH release in samples functionalized with GO (both control and test) compared to control was evidenced, even if it was not statistically significant (Figure 7).

**Figure 8:**
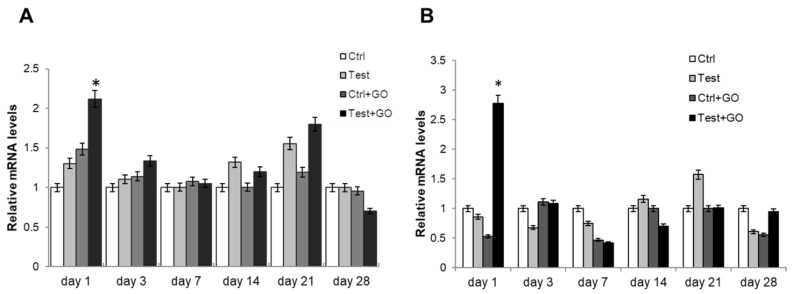
Relative gene expression of (**A**) TGFβ and (**B**) BMP2 in DPSCs cultured on Ti discs for 1, 3, 7, 14, 21, and 28 days. Data are expressed as relative to control (calibrator sample, defined as 1). Values represent the means ± SD. *Y*-axis, fold change. The most representative of three separate experiments is shown.

**Figure 9:**
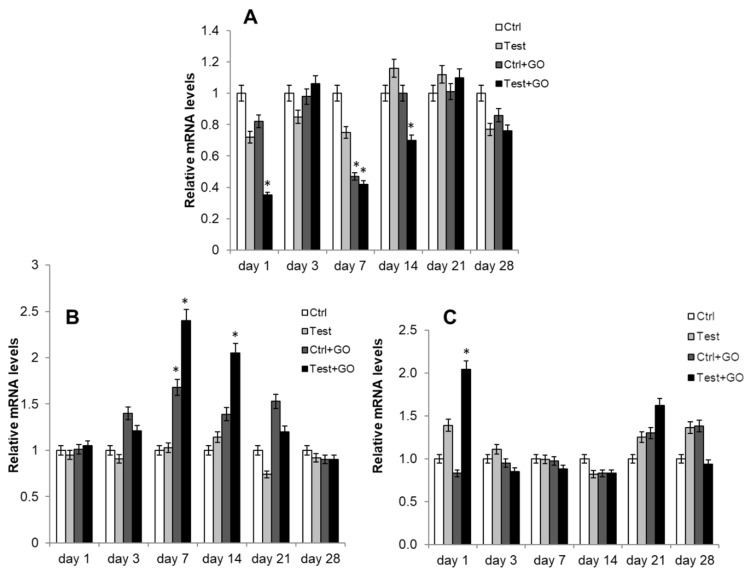
Relative gene expression of (**A**) RUNX2, (**B**) SP7, and (**C**) COL1A1 in DPSCs cultured on Ti discs for 1, 3, 7, 14, 21, and 28 days. Data are expressed as relative to control (calibrator sample, defined as 1). Values represent the means ± SD. *Y*-axis, fold change. The most representative of three separate experiments is shown. *RUNX2, day 1: test + GO vs. control, test, control + GO *p* < 0.05; day 7: control + GO vs. control *p* < 0.05, test + GO vs. control *p* < 0.05; day 14: test + GO vs. control, test, control + GO *p* < 0.05. *SP7, day 7: control + GO, vs. control, test *p* < 0.01; test + GO vs. control, test *p* < 0.01; day 14: test + GO vs. control, test *p* < 0.01. *COL1A1, day 1: test + GO vs. control, test *p* < 0.01.

The differentiation process of DPSCs towards the osteoblastic phenotype was evaluated by measuring the gene expression of different early and late osteoblastic markers through real-time RT-PCR. TGFβ gene expression, whose pathway activates RUNX2, showed a statistically significant increase in test + GO sample compared to control and test, after one day of culture (Figure 8A). BMP2 gene expression also was significantly superior in the test + GO sample at the same timepoint with respect to all other experimental points (Figure 8B). Conversely, RUNX2 gene expression was significantly lower in the test + GO sample after one day of culture compared to all the other samples, while after seven days of culture, a statistically significant reduced gene expression could be recorded in both control + GO and test + GO samples. After 14 days of culture, a reduced RUNX2 gene expression was detected only in the test + GO sample (Figure 9A). An increase of SP7 gene expression was found after seven days of culture in control + GO and test + GO samples compared to control and test, whereas an increase in mRNA level was recorded only in the test + GO sample after 14 days of culture (Figure 9B). COL1A1 gene expression increased (i.e. the increase is statistically significant) in the test + GO sample, compared to control and test, after one day of culture (Figure 9C).

**Figure 10:**
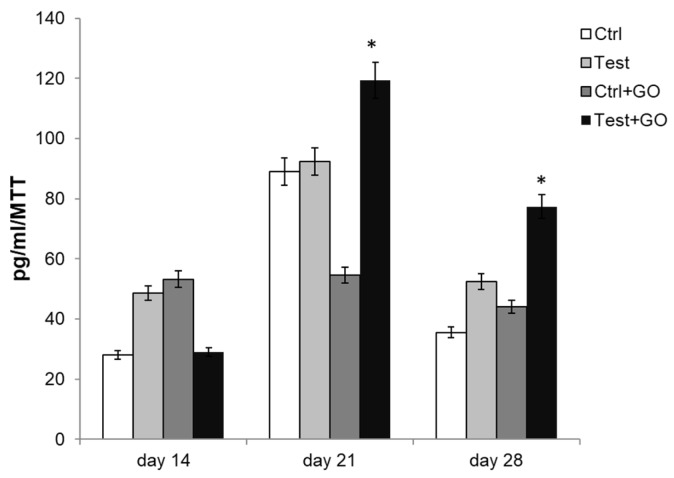
ELISA assay for PGE2 secretion of DPSCs cultured on Ti discs for 14, 21, and 28 days. Secretion levels are reported as picograms per milliliter per MTT optical density (OD) values. The results are the mean ± SD of three samples from three different experiments. * Day 21 and day 28: test + GO vs. control, test, control + GO *p* < 0.05.

Finally, PGE2 secretion level was measured through ELISA assay. PGE2 was increased in test + GO, with respect to all other experimental samples after both 21 and 28 days of culture (Figure 10).

## 4. Discussion

Dental implants placement is still the first-choice treatment for solving the edentulous patients’ issues. Due to its chemical and physical properties, titanium is nowadays considered the most biocompatible material for making dental implants. Moreover, its capability to form a thin layer of TiO_2_ on the surface improves biocompatibility by avoiding the corrosion process and ions release [34]. Several studies demonstrated that surface modifications in dental implants are able to further ameliorate their integration process into the host bone tissue. In particular, a previous study performed in our laboratory demonstrated that a sandblasted and acid-etched surface further treated with inorganic ions guarantees an improvement in cell viability, a low inflammatory response, a faster promotion of the osteoblastic differentiation, a higher biocompatibility, and a better tolerability by both human osteoblasts and DPSCs, compared to a simply sandblasted and acid-etched titanium surface [6,7]. For this reason, we decided to compare, in the present study, only machined, i.e. not treated, control, and test (i.e. sandblasted and acid-etched) discs. We evaluated whether the functionalization of control and test Ti surfaces with GO could improve the titanium biocompatibility and the DPSCs differentiation towards the osteoblastic lineage. The idea to covalently functionalize the Ti discs, rather than simply physically load GO on Ti surfaces, derives from the aim of preparing a material that did not leak GO in the neighboring tissue. As a matter of fact, in a previous study on porcine bone granules physically coated with GO, aggregated GO were evidenced in the soft tissue after three months deposition in the animal, despite that the same material did not show any evidences of GO leakage after seven days of dip in aqueous solution or PBS [20]. The covalent functionalization ensures that excess GO, not properly bound to the surface, is washed out from the material.

From Raman and SEM data, it was possible to highlight that the optimized protocol of functionalization allows a homogenous functionalization of the titanium discs surface without altering the corresponding surfaces roughness, as shown by AFM measurements.

DPSCs were selected because of their extensive differentiation ability and the easy access to the site of collection [31]. As demonstrated by MTT and LDH test, GO enrichment did not produce a cytotoxic response. In fact, even if at the early stage of culture a general high cytotoxic response is identified in all tested samples, there are no significant differences among the different surfaces. This lead to the hypothesis that this initial high cytotoxic response is attributable to the early phases of the adhesion process, due to the fact that the titanium disc is not a cell culture-treated surface. This hypothesis, already evidenced in other experimental models involving cultures on biomaterials [35], is also confirmed by the observed progressive decreasing trend over culture time, which clearly highlights an appreciable and increasing surface biocompatibility for all tested surfaces. Moreover, the GO enrichment was able to promote the cell growth when a confluence condition had not been achieved yet, thus leading to the hypothesis that the GO coating represents an additional factor responsible for further enhancement of the titanium biocompatibility.

In this context, SEM analyses showed that after 14 days of culture, a well-organized collagen fibers network (which is essential for the further deposition of bone minerals [36]) is detected only on test + GO samples.

The GO capability to promote DPSCs osteogenic differentiation is further confirmed as BMP2, a potent early osteogenic marker that stimulates maturation of mesenchymal stem cells into osteoblasts [37,38], shows significantly increased expression levels only in test + GO samples after one day of culture. 

As expected, considering that the combined activity of TGFβ and BMP pathways lead to positive regulation of RUNX2 transcription factor [39], we observed also that TGFβ gene expression increased after one day of culture in the test + GO sample. This result agrees with previous studies reporting that TGFβ/BMP signaling activation is fundamental during guided bone regeneration [40].

RUNX2 is a master transcription factor that regulates the expression of several osteoblast marker genes, such as collagen type I alpha I (Col1a1) and osterix (SP7) [41]. It functions as a mediator in osteoblast differentiation, in a stage-dependent way. In fact, during the early phases, RUNX2 promotes the transformation from osteoprogenitor cells to preosteoblasts, while it inhibits the process at later stages [42]. In our experimental model, this trend in RUNX2 gene expression was maintained only in test + GO samples. In fact, after three days of culture, RUNX2 expression raised in control + GO and in test + GO, suggesting that both the surfaces were able to trigger the differentiation process. However, the subsequent reduction of RUNX2, that is required by the osteogenic differentiation progress, was recorded after 7 and 14 days of culture only in the test + GO sample. 

RUNX2 coordinates with SP7, a downstream effector of RUNX2, for the improvement of the osteoblastic differentiation process. Indeed, it has been shown that osterix -/- mice showed complete lack of osteoblasts [43]. Our model clearly revealed a SP7 increase in test + GO following RUNX2 upregulation, thus confirming that osteoblastic differentiation progresses only when DPSCs were cultured on test + GO surface. 

Moreover, a very early increase in COL1A1 gene expression was also detected on test + GO. This is in agreement with the findings of Lagenbach and colleagues [44], which underlined that RUNX2 is tightly regulated by upstream factors and cofactors, such as COL1A1, which is able to activate RUNX2 by phosphorylation via MAPK signalling. 

Despite the fact that high secretion of prostaglandins is commonly responsible for an inflammation event occurrence, PGE2 behave as positive mediators of the osteoblastic differentiation process [45,46]. Indeed, we noticed that the decoration of the test surfaces with GO leads to an increase in PGE2 secretion level compared to all the other surfaces, thus confirming the hypothesis of a more efficient osteoblastic differentiation when GO is applied on test discs. Indeed, this effect is not detected neither on test without GO decoration nor on control + GO surfaces, suggesting that the ideal combination for a better and faster osteoblastic differentiation is represented by test + GO samples. Recently, Conserva and colleagues underlined the pivotal role exerted by immunomodulatory capabilities of the mesenchymal stem cells when the interaction biomaterial/cell is evaluated, evidencing that the implant integration with the surrounding bone tissue is often facilitated when signaling pathways, leading to modulation of inflammatory cascades, are activated [47]. In particular, it was demonstrated that a PGE2 secretion down-regulation in adult stem cells is able to significantly impair their immunomodulatory strength [48]. In light of this evidence, our data regarding PGE2 secretion lead us to argue that GO is able to create an appreciable osteo-immunomodulatory environment, as already reported elsewhere [49] and that GO immunomodulation is only appreciable with GO-functionalized test surfaces. However, further investigations, focused on the recruitment of molecular pathways leading to the decline of immunoregulatory capability, will needed to better and deeper clarify the possible role of GO as an immunomodulatory agent.

## 5. Conclusions

Titanium surfaces were activated with piranha solution or by using a UV/ozone lamp and coated with GO via covalent bond formation. The GO functionalization procedure was effective on both control and test surfaces. No significant difference in cell viability and toxicity, compared to the untreated Ti discs, was found in the functionalized samples. The experimental evidence shows that test + GO titanium discs have outstanding capabilities in promoting DPSCs differentiation into the osteoblastic cell lineage: the gene expression, particularly that of TGFβ and BMP2 at early stage, leads to positive regulation of RUNX2 transcription factor at day three. Furthermore, the subsequent SP7 increase in test + GO following RUNX2 upregulation confirmed the osteoblastic differentiation progress only when DPSCs were cultured on test + GO surface. Moreover, the increased secretion of PGE2 also evidences a possible immunomodulatory role for GO. These results promote the GO-functionalization technique described herein for the production of novel dental implant materials, displaying an improved integration with the host tissue.

## Figures and Tables

**Table 1:** TaqMan probes and PCR primers used in the experiments.

TaqMan Probes	Gene Detected
Hs00998133_m1	TGFβ
Hs00154192_m1	BMP2
Hs00231692_m1	RUNX2
Hs01866874_s1	SP7
Hs00164004_m1	Collagen type I
Hs99999905_m1	GAPDH

**Table 2:** Mean values of the absolute values of: the surface high deviations (R_a_), the roughness least square value (R_q_), the maximum perpendicular distance between the highest and lowest points in the image following the plane fit (R_max_), the root-mean square of the surface slope (S_dq_), and the percentage of additional surface area contributed by the new texture as compared to an ideal plane of the same region (S_dr_), obtained from the five individual measurements using Multimode 8 Bruker AFM and nanoscope analysis software.

Sample	R_a_ (nm) (± SD)	R_q_ (nm) (± SD)	R_max_ (nm) (± SD)	S_dq_ (deg) (± SD)	S_dr_ (%) (± SD)
control	48.0 ± 5.5	59.5 ± 6.9	378.2 ± 54.5	11.4 ± 4.1	1.7 ± 0.7
control + GO	40.0 ± 8.3	45.9 ± 7.6	335 ± 118	7.8 ± 0.8	0.96 ± 0.18
test	196.2 ± 48	247.4 ± 56.4	1643 ± 514	30.5 ± 7.5	17.1 ± 11.4
test + GO	235.4 ± 100.5	289.8 ± 121.1	1546 ± 484	20.3 ± 3.8	6.8 ± 2.4

## Supplementary Materials

The following are available online at https://www.mdpi.com/2079-4991/10/4/654/s1, Table S1: R_a_, R_q_, R_max_, S_dq,_ and S_dr_ values obtained from five individual measurements, using Multimode 8 Bruker AFM and nanoscope analysis software.
