# Supplementary material for: Osteoblastic Differentiation on Graphene Oxide-Functionalized Titanium Surfaces: An In Vitro Study
Source: Nanomaterials (Basel). 2020 Apr 1;10(4):654. doi: 10.3390/nano10040654 (PMC7221922; doi:10.3390/nano10040654)
Supplement: Supplementary file 1 [file nanomaterials-10-00654-s001.pdf]

Osteoblastic Differentiation on Graphene Oxide-Functionalized Titanium Surfaces: An In Vitro Study

Roberta Di Carlo [1], Antonello Di Crescenzo [2], Serena Pilato [2], Alessia Ventrella [2], Adriano Piattelli [1], Lucia Recinella [2], Annalisa Chiavaroli [2], Silvia Giordani [3], Michele Baldrighi [4], Adalberto Camisasca [3], Barbara Zavan [5], Mirella Falconi [6], Amelia Cataldi [2], Antonella Fontana[2,*] and Susi Zara [2]

[1] Department of Medical, Oral and Biotechnological Sciences, University "G. d'Annunzio" of Chieti-Pescara, via dei Vestini 31, 66100 Chieti, Italy; roberta.dicarlo@unich.it (R.D.C.); adriano.piattelli@unich.it (A.P.)

[2] Department of Pharmacy, University "G. d'Annunzio" of Chieti-Pescara, via dei Vestini 31, 66100 Chieti, Italy; a.dicrescenzo@unich.it (A.D.C.); serena.pilato@unich.it (S.P.); alessia.ventrella@unich.it (A.V.); lucia.recinella@unich.it (L.R.); annalisa.chiavaroli@unich.it (A.C.); amelia.cataldi@unich.it (A.C.); susi.zara@unich.it (S.Z.)

[3] School of Chemical Sciences, Dublin City University, Glasnevin, D09 E432 Dublin 9, Ireland; silvia.giordani@dcu.ie (S.G.); adalberto.camisasca@dcu.ie (A.C.)

[4] Nano Carbon Materials, Italian Institute of Technology, via Morego 30, 16163 Genova, Italy; michele.baldrighi@gmail.com

[5] Medical Science Department, University of Ferrara, via Aldo Moro 8, 44121 Ferrara, Italy; zvnbbr@unife.it

[6] Department of Biomedical and Neuromotor Sciences, University of Bologna, via Irnerio 48, 40126 Bologna, Italy; mirella.falconi@unibo.it

* Correspondence: fontana@unich.it; Tel.: +39-0871-355-4790

SUPPLEMENTARY MATERIAL

Table S1. R_a, R_q, R_{max}, S_{dq} and S_{dr} values obtained from five individual measurements using Multimode 8 Bruker AFM and Nanoscope analysis software.

Sample	R_a (nm)	R_q (nm)	R_{max} (nm)	S_{dq} (deg)	S_{dr} (%)
Ctrl 1	52.6	63.9	458	9.6°	1.4%
Ctrl 2	46.1	56.3	312	9.2°	1.3%
Ctrl 3	54.9	69.4	380	18.3°	2.6%
Ctrl 4	42.1	53.5	348	7.9°	0.9%
Ctrl 5	44.5	54.2	393	12.0°	2.2%
Ctrl + GO 1	42.1	53.5	348	7.9°	1.0%
Ctrl + GO 2	48.6	41.5	535	7.6°	0.9%
Ctrl + GO 3	43.9	53.0	282	8.9°	1.2%
Ctrl + GO 4	38.9	45.8	244	8.1°	1.0%
Ctrl + GO 5	26.6	35.6	264	6.6°	0.7%
Test 1	260	317	1844	32.1°	16.4%
Test 2	126	163	932	26.1°	10.9%
Test 3	209	272	1996	43.1°	36.9%
Test 4	203	250	1288	25.3°	10.2%
Test 5	183	235	2153	26.1°	10.9%
Test + GO 1	135	172	1127	14.5°	3.3%
Test + GO 2	188	237	1272	18.9°	5.7%
Test + GO 3	265	317	1691	20.7°	6.9%
Test + GO 4	395	486	2329	23.7°	9.2%
Test + GO 5	194	237	1313	23.5°	8.9 %
